# A cross-sectional study of sleep, mood, well-being, motivations, and perceived support in Ukrainian veterans and active-duty military personnel with disability, and their supporters, preparing for a sporting event

**DOI:** 10.3389/fpsyg.2023.1089735

**Published:** 2023-03-23

**Authors:** Claire L. Castle, Nikki Heinze, Renata S. M. Gomes

**Affiliations:** ^1^BRAVO VICTOR, London, United Kingdom; ^2^School of Music, Faculty of Arts, Humanities and Cultures, University of Leeds, Leeds, United Kingdom; ^3^Northern Hub for Veterans and Military Families Research, Department of Nursing, Midwifery and Health, Faculty of Health and Life Sciences, Northumbria University, Newcastle upon Tyne, United Kingdom

**Keywords:** competition, sports, disability, veteran, sleep, military, well-being, mood

## Abstract

**Purpose:**

The benefits of sports and exercise to the lives and rehabilitative journeys of military veterans with disabilities is increasingly well-documented but veteran sporting events remain underexplored. Addressing this topic, the current article seeks to provide insight into the health and well-being of Team Ukraine during a 5-week preparatory camp in the UK before attendance at the 2022 Warrior Games.

**Materials and methods:**

Two surveys were run, one toward the beginning and one toward the end of the camp. Eighteen of the 55 veterans and serving personnel (with disability), support staff, and family members in attendance responded to both surveys. Data on sleep, mood, and competition-related emotions, motivations for participation and perceived support were gathered. Data were analysed descriptively, and sleep, mood, and competition-related emotion responses were categorised to explore improvements, maintenance, or worsening in these areas.

**Results and conclusion:**

Sleep, mood, and competition-related emotions were relatively stable, although sleep duration was low, and there were some increases in daytime dysfunction, anxiety, nervousness, and feeling tense. Family was the most important source of support and representation of one’s country and raising awareness of Ukraine’s circumstances were the most important motivational factors. Findings offer insight into not only the health and well-being experiences associated with participation in this disabled veteran sporting event, but also the important role played by this event in meeting collective goals relating to this unique time in Ukraine’s history.

## Introduction

Research highlights the benefits of sports and physical activity to the lives and rehabilitative journeys of military veterans (Caddick and Smith, 2014). Veterans with traumatic injuries or chronic illness have noted the motivating nature of sports, with a focus on capacity and success rather than illness or injury, and the availability of immediate short- and long-term feedback when participating in sports (Brittain et al., 2022). Participation in sports and physical activity has been associated with improved subjective (e.g., active coping and social participation), psychological (e.g., increased determination, focus on ability, improved self-concept), and social well-being among combat veterans (Caddick and Smith, 2014). Furthermore, physical benefits such as increased mobility, strength, and aerobic capacity (Addison et al., 2019; Briggs and Oursler, 2021), weight loss and increased fitness (Brittain et al., 2022), and reductions in the symptomology of mental health conditions such as post-traumatic stress disorder (PTSD) have been identified in veterans with disabilities, traumatic injuries or chronic illness (Walter et al., 2021). Outdoor and nature-based activities, such as fishing, water and snow sports, riding, and archery, have been found to provide a wide range of benefits to veterans, including facilitating positive mood and establishing new coping methods through motivation and challenge (Bennett et al., 2017; Craig et al., 2020), opportunities for social bonding (Lundberg et al., 2016), as well as reductions in PTSD symptomology (Gelkopf et al., 2013; Crawford, 2016; Wheeler et al., 2020).

Literature has also explored the experiences, benefits, and challenges of participating in sporting events for veterans with disabilities (Roberts et al., 2019, 2021). Participants at the National Veterans Wheelchair Games (NVWG) and Winter Sports Clinic (NVWSC, both held in the USA) reported increased mobility and acceptance of their disability (Sporner et al., 2009). Invictus Games competitors have cited improvements in physical and mental health and performance, and social interactions (Roberts et al., 2021), opportunities to develop goal-setting and teamwork skills, and a chance to reconnect with a military identity (Brittain et al., 2022). Research has also considered the experiences of participants of The Warrior Games (run by the USA’s Department of Defence). Peterson et al. (2017) found that time spent participating in organised sporting events impacted on skills relating to performance strategy, with those who had participated for over a year demonstrating greater skills relating to self-talk, goal setting and activation prior to competition than those who had less than 1 year of experience. Similarly, Laferrier et al. (2017) found that registered athletes at the NVWSC, the Warrior Games, and the NVWG, had significantly higher self-esteem scores than those not involved in sports and exercise, with this score also being significantly higher immediately following participation in a sporting event compared to 1 and 3 months after an event. Participation in sports and exercise was also associated with significantly lower scores on depression measures, and higher post-traumatic growth and quality of life scores. Those who had been participating in sports and exercise for longer periods also scored significantly higher on measures compared to those who had participated for less time. However, negative experiences have also been identified, such as a perceived lack of goals after the games, “post-games blues,” and experiences of stress before and during the games (Roberts et al., 2019, 2021). Veteran sporting events remain an underexplored topic, and there has been limited research in this area in recent years due to the cancellation of events during the COVID-19 pandemic.

In 2022, the United States Department of Defence invited Ukrainian veterans and personnel injured in the current conflict to compete alongside American and Canadian service teams in the Warrior Games (“the Games”) held in Florida. The Games were attended by 61 Ukrainian competitors and support staff, including a small number of widows and children of deceased personnel; 55 of these individuals attended a training camp in the United Kingdom (UK) to prepare for the Games, hosted by Blind Veterans UK. Veteran participants were airlifted directly from an active warzone and military duties. The camp consisted of an intensive 5-week schedule of training sessions, psychotherapy sessions, and recreational trips. This camp provided an opportunity to gain unique insight into experiences associated with Team Ukraine’s preparations for the Games. The current study aimed to explore aspects of the team’s health and well-being during this time, and factors that might impact on their experiences of preparing for the Games. Addressing this objective, the current article answers the following questions:

(1) How, if at all, did mood, well-being, sleep, and competition-related emotions change in members of Team Ukraine over the course of their UK-based preparations for the Games?

(2) What motivations for participation in the Games were most important to members of Team Ukraine?

(3) What sources of support were perceived as most important to Team Ukraine during their preparations for the Games in UK?

## Materials and methods

Two online surveys were conducted, one at the start and another at the end of the camp, to gather quantitative data relating to demographics, health and well-being, motivations for participation, and perceived support. Open-ended questions collected qualitative data on participants’ preparations, motivations, goals, and achievements. The current article reports quantitative findings only. Run as part of the evaluation of the training camp, the current study did not require approval from an ethics panel; this was confirmed by the Chair of the Medical Sciences Interdivisional Research Ethics Committee at the University of Oxford.

### Participants and recruitment

A total of 55 veterans, current serving personnel, widowed spouses and support staff were airlifted out of Ukraine by the British military to attend the training camp in the UK to prepare for the Games. All 55 members of Team Ukraine who attended the training camp were invited to take part in the surveys. Participation was voluntary. The Ukrainian team leadership notified the team of the study. Participants were able to access the surveys using a link or QR code in a flyer provided in English and Ukrainian. The team leadership also shared a link *via* a private communication app accessible only to the team. A member of the research team was present throughout the camp to answer any questions.

### Materials

Surveys 1 and 2 gathered data on sleep, mood, well-being, and competition-related emotions. Survey 1 (S1) explored age, type of disability, role in team, participation in different sporting events, previous sporting competition experience and motivations for taking part in the Games. Survey 2 (S2) assessed gender and asked participants to reflect on the importance of different sources of support during their preparations.

Questionnaires for both surveys were designed to minimise the impact of the research on the team’s training schedule and preparations for the Games. As such, relevant individual items were selected from existing scales or surveys or developed for the survey. Sleep was assessed using four items from the Pittsburgh Sleep Quality Index (PSQI) (Buysse et al., 1989). The full PSQI scale score has an internal consistency of α = 0.83 and a test–retest reliability of *r* = 0.87 (Buysse et al., 1989). The items selected in the current study explored subjective sleep quality (one item), sleep duration (one item), and daytime dysfunction (two items). Ukrainian translations were obtained from the MAPI Research Trust.

Mood was assessed using 10 items from the Ukrainian version of the European Social Survey (2010) (ESS). The items explored how often people felt, for example, “depressed” or “happy” in the past week. Responses were selected from a scale ranging from “None or almost none of the time” to “All or almost all of the time.” One item, “Felt guilty,” was added to acknowledge the guilt that those involved in active duties might experience when absent from their unit and comrades in Ukraine.

Personal well-being was assessed using two items from the ONS-4: life satisfaction and feeling like life is worthwhile (Office for National Statistics, 2018). The ONS-4 have been assessed through multiple waves of cognitive interviews and are regularly included in large general population surveys in the UK. The items require participants to give a response on a scale of 0 to 10 (where 0 is “not at all” and 10 is “completely”) to indicate the extent to which they feel satisfied with life and like life is worthwhile. ONS-4 items relating to anxiety and happiness were omitted to avoid duplication with ESS items.

Competition-related emotions were assessed using items addressing current anxiety relating to the upcoming competition from all three subscales (Worry, Concentration Disruption, Somatic Trait Anxiety) of The Sport Anxiety Scale-2 (SAS-2) (Smith et al., 2006). Participants were asked to rate the extent to which they felt a certain way from 1, “not at all” to 4, “very much.” Additional items, “I feel… confident/excited/supported” and “I am looking forward to competing” were included to explore positive emotions. Total score alpha coefficients exceeding 0.89 have been reported across all age groups (including children and adults) for the SAS-2 (Smith et al., 2006), suggesting internal consistency. The SAS-2 has been found to be reliable, gender invariant, and to have strong construct validity (Madrigal et al., 2018), although, it has not yet been validated with a Ukrainian sample. In the current study the full sub-scales were not employed; only items dealing with current, state, anxiety (e.g., “I feel nervous”) and not hypothetical experiences (e.g., “My mind wanders during sport competition”) were included. This reflected the need to minimise participant burden and training disruption.

A question was developed for S1 to assess participants’ motivations for their involvement in the Games. Participants were asked to rate how important a range of 14 possible reasons, such as spending time with other veterans or representing their country, were in their decision to take part in the Games, on a scale ranging from “extremely important” to “not at all important.” The importance of different sources of support was assessed with a question developed for S2. This question asked participants to rate on a scale ranging from 1, “not at all” to 6, “completely,” how important nine different sources of support (e.g., their unit, family, or fellow competitors) were to them during their preparations. All questions included a “Prefer not to say” response option.

Except for questions where Ukrainian translations were available, the questionnaires were translated into Ukrainian by a professional translation agency and checked by a member of Team Ukraine.

### Procedure

S1 was run the second week of Team Ukraine’s time in the UK (22nd–26th July) and S2 ran during their final days in the UK (14th–18th August). Paper and English versions were available, but all participants completed the surveys online in Ukrainian. The surveys were delivered using the online survey platform SmartSurvey. An accessible survey template was used to ensure accessibility for participants with visual impairment. Participants were provided with an information sheet and required to provide informed consent at the beginning of S1. Participants reconfirmed their consent to participate before completing S2.

### Analysis

Due to the selection of individual items from scales, no full-scale scores were computed. Frequencies of responses are reported, along with descriptive statistics such as mean, standard deviation and range where relevant. These provide an overview of sample demographics, sleep, mood, well-being, and competition-related emotions at the two timespoints, and data relating to motivations for participation and perceived sources of support. Change scores were created to explore if respondents’ sleep, mood, well-being, and competition-related emotions had improved, stayed the same, or got worse. Due to the small sample size, comparative statistical analysis and subgroup analysis was not undertaken.

## Results

### Demographics and upcoming sporting events

After excluding 2 partial responses, a total of 18 of the 55 camp attendees completed both surveys (36% response rate). Table 1 shows sample demographics, and the events competitors participated in. Respondents were aged 25–56 years (*M* = 38.38 years, *SD* = 9.67). Six respondents were female (33%). The sample consisted of 3 military personnel on active duty (16.7%), 4 veterans who had re-joined active duty (22.2%), 4 veterans (22.2%) (collectively referred to throughout as the “veteran” group), 4 members of support staff (22.2%), 2 family members who had taken the place of fallen team members (11.1%), and one coach (5.6%). Respondents reported an average of 1.61 disabilities (*SD* = 1.58, range: 0–4), most commonly disabilities affecting mobility (*n* = 7). Seven also reported “Other” disabilities, whilst 5 reported a visual impairment (VI), 4 a chronic pain condition, 3 a hearing impairment, and 3 limb loss. Six participants reported multiple disabilities. Mental health conditions were evident but less prevalent; PTSD was most common (*n* = 4), followed by emotional or behavioural difficulties (*n* = 3). Health data was self-reported and may not have reflected clinical diagnoses. Respondents participated in an average of 3.83 (*SD* = 4.33) and a maximum of 17 sporting events.

**TABLE 1:** Sample demographics and sports to be competed in at the Warrior games.

		*M* (*SD*, range)	% (*n*)
Age		38.83 (9.67, 25–56)	
Sex	Female		33.3 (6)
	Male		66.7 (12)
Disabilities	Number	1.61 (1.58, 0–4)	
	VI		27.8 (5)
	Hearing		16.7 (3)
	Limb loss		16.7 (3)
	Mobility		38.9 (7)
	Pain		22.2 (4)
	Other		38.9 (7)
Mental health conditions	Number	0.44 (0.70, 0–2)	
	Anxiety		–
	PTSD		22.2 (4)
	Depression		5.6 (1)
	Emotional/behavioural difficulties		16.7 (3)
	Other mental health conditions		–
	Prefer not to say		5.6 (1)
Role in team	Athlete (active-duty military member)		16.7 (3)
	Athlete (veteran, who re-joined active duty)		22.2 (4)
	Athlete (veteran)		22.2 (4)
	Coach		5.6 (1)
	Family		11.1 (2)
	Support		22.2 (4)
Sports events	Number	5.5 (4.18, 2–17)	
Team sports:	Volleyball		45.5 (5)
	Basketball		27.3 (3)
	Rugby		9.1 (1)
	Powerlifting		45.5 (5)
Archery:	Team		36.4 (4)
	Individual		72.7 (8)
Track:	100 m		9.1 (1)
	1500 m		18.2 (2)
	Relay		9.1 (1)
Field:	Discus		18.2 (2)
	Shot put		18.2 (2)
Cycling:	Road		18.2 (2)
	Time		9.1 (1)
Rowing:	1-min		27.3 (3)
	4-min		36.4 (4)
Swimming:	50 m freestyle		36.4 (4)
	100 m freestyle		18.2 (2)
	Backstroke		27.3 (3)
	Breaststroke		9.1 (1)
Air rifle:	Prone		9.1 (1)
	Standing		18.2 (2)

### Sleep and mood

**FIGURE 1:**
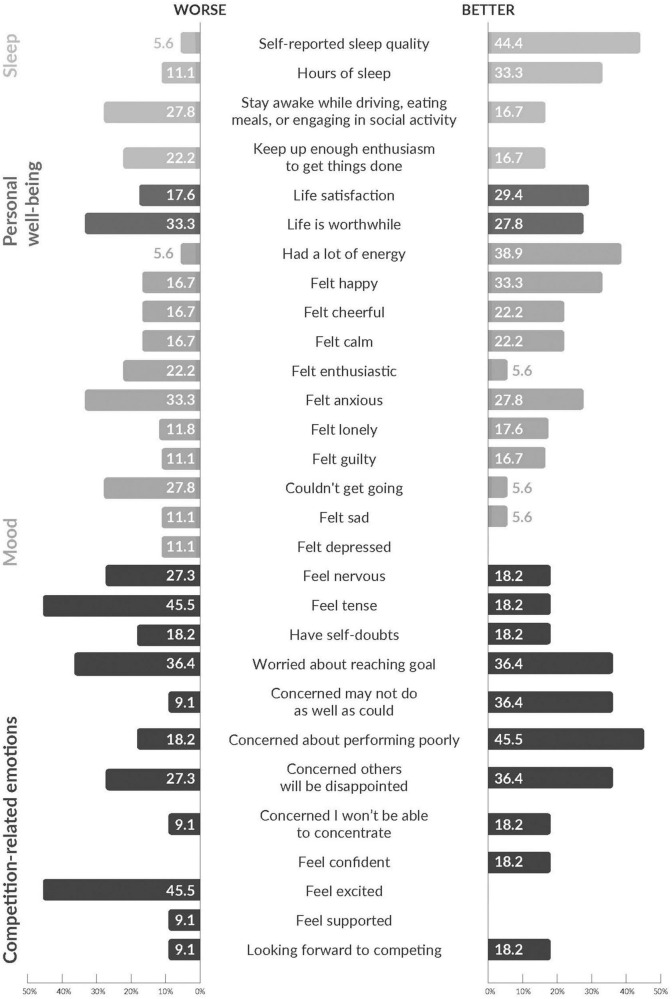
Categorical change in mood, sleep, and competition-related emotions.

For most participants sleep, life satisfaction, life being worthwhile, and mood did not change between the surveys (Table 2). There was a small increase in mean sleep duration (from 6.18 to 6.67 h) (Table 3), and minimum sleep duration (from 3 to 4 h). However, participants were more likely to report poorer sleep duration at S2 than improved sleep duration. The proportion of participants who rated their sleep quality as “fairly” or “very good” increased from 50.0% at S1 to 83.3% at S2, but the number of participants who reported “very bad” sleep also increased from 1 to 2 (Table 4). In contrast, instances of daytime dysfunction, including difficulties staying awake during the day and keeping up enthusiasm to get things done, appeared to increase at S2 (Table 4). Whilst sleep quality and duration improved for a greater number of individuals than it worsened, the 2 daytime dysfunction items worsened for a greater number of individuals than the number for whom it improved (Figure 1).

**TABLE 2:** Categorical change in mood, sleep, and competition-related emotions.

	Better	Same	Worse
	**%**	**%**	**%**
**Personal well-being (*n* = 18)**			
Life satisfaction	27.8	50	16.7
Life is worthwhile	27.8	38.9	33.3
**Mood (*n* = 18)**			
Felt cheerful	22.2	61.1	16.7
Felt depressed	–	83.3	11.1
Felt happy	33.3	50	16.7
Felt lonely	16.7	66.7	11.1
Felt sad	5.6	77.8	11.1
Couldn’t get going	5.6	61.1	27.8
Had a lot of energy	38.9	50	5.6
Felt anxious	27.8	33.3	33.3
Felt calm	22.2	55.6	16.7
Felt enthusiastic	5.6	72.2	22.2
Felt guilty	16.7	66.7	11.1
**Sleep (*n* = 18)**			
Self-reported sleep quality	44.4	44.4	5.6
Hours of sleep	33.3	50	11.1
Stay awake during activities	16.7	38.9	27.8
Keep up enthusiasm to get things done	16.7	55.6	22.2
**Competition-related emotions (*n* = 11, athletes only)**			
Feel nervous	18.2	54.5	27.3
Feel confident	18.2	81.8	–
Feel tense	18.2	27.3	45.5
Feel excited	–	54.5	45.5
Feel supported	–	81.8	9.1
Have self-doubts	18.2	63.6	18.2
Worried about reaching goal	36.4	27.3	36.4
Looking forward to competing	18.2	63.3	9.1
Concerned may not do as well as could	36.4	45.5	9.1
Concerned about performing poorly	45.5	27.3	18.2
Concerned others will be disappointed	36.4	36.4	27.3
Concerned I won’t be able to concentrate	18.2	63.6	9.1

Missing responses and “Prefer not to say” responses are not shown. Percentages refer to the *n* shown for each section. Percentages may not add up to 100% due to rounding, missing responses and “Prefer not to say” responses.

**TABLE 3:** Sleep, personal well-being, and sources of support in Survey 1 and Survey 2.

		Survey 1		Survey 2	
		***n***	***M*** **(*SD*)**	***n***	***M*** **(*SD*)**
Sleep	Sleep duration	17	6.18 (1.59)	18	6.67 (1.14)
Personal well-being	Life satisfaction	17	7.71 (2.47)	18	8.17 (1.82)
	Life being worthwhile	18	8.56 (1.5)	18	8.33 (1.94)
Sources of support	Family			17	5.35 (1.54)
	Sports coaches and trainers			16	5.25 (1.24)
	Friends			17	4.88 (1.54)
	Hosts in the United Kingdom			14	4.43 (1.56)
	Military unit			12	4.17 (1.64)
	Physiotherapist or other rehabilitative support			14	3.93 (1.98)
	Fellow competitors			13	3.92 (1.55)
	Sports psychologist			15	3.67 (2.09)
	Religion or spirituality			16	3.63 (2.22)

*n* = valid responses excluding missing responses and “Prefer not to say” responses. M, mean; SD, standard deviation.

**TABLE 4:** Self-reported sleep quality and daytime dysfunction in Survey 1 and Survey 2.

		Survey 1	Survey 2
**Variable**	**Response options**	***n*** **(%)**	***n*** **(%)**
Sleep quality	Very good	–	2 (11.1)
	Fairly good	9 (50.0)	13 (72.2)
	Fairly bad	7 (38.9)	1 (5.6)
	Very bad	1 (5.9)	2 (11.1)
Staying awake	Not during the past month	6 (33.3)	5 (27.8)
	Less than once or twice	4 (22.2)	6 (33.3)
	Once or twice a week	5 (27.8)	7 (38.9)
	3 or more times a week	–	–
	Prefer not to say	3 (16.7)	–
Enthusiasm	No problem at all	7 (38.9)	7 (38.9)
	Only a very slight problem	7 (38.9)	6 (33.3)
	Somewhat of a problem	2 (11.1)	5 (27.8)
	A very big problem	1 (5.6)	–
	Prefer not to say	1 (5.6)	–

*n* = frequency of respondents who gave this answer, %, proportion of respondents who gave this answer based on full sample. Missing responses are not shown. Percentages may not add up to 100% due to rounding, missing responses, and “Prefer not to say” responses.

Mean life satisfaction increased from 7.71 (*SD* = 2.47) to 8.17 at S2 (*SD* = 1.82) but mean life being worthwhile decreased slightly from 8.56 (*SD* = 1.50) to 8.33 (*SD* = 1.94). As seen in Figure 1, more people felt cheerful, happy, full of energy and calm more often, and lonely and guilty less often, at S2. However, more people also felt depressed, sad, that they couldn’t get going and anxious more often, and enthusiastic less often. For instance, while 61.1% felt happy at least most of the time at S1, this increased to 88.9% at S2. A greater number of respondents felt anxious “some of the time” at S2, and a smaller number felt anxious “none or almost none of the time” (Table 5).

**TABLE 5:** Mood in Survey 1 and Survey 2.

		(Almost) all of the time	Most of the time	Some of the time	(Almost) none of the time
		***n*** **(%)**	***n*** **(%)**	***n*** **(%)**	***n*** **(%)**
Felt cheerful	S1	7 (38.9)	9 (50.0)	2 (11.1)	–
	S2	9 (50.0)	6 (33.3)	3 (16.7)	–
Felt depressed	S1	–	–	4 (22.2)	14 (77.8)
	S2	–	–	6 (33.3)	11 (61.1)
Felt happy	S1	4 (22.2)	7 (38.9)	6 (33.3)	1 (5.6)
	S2	2 (11.1)	14 (77.8)	1 (5.6)	1 (5.6)
Felt lonely	S1	–	1 (5.6)	6 (33.3)	11 (61.1)
	S2	–	1 (5.6)	5 (27.8)	11 (61.1)
Felt sad	S1	–	–	11 (61.1)	7 (38.9)
	S2	–	–	11 (61.1)	6 (33.3)
Could not get going	S1	–	–	4 (22.2)	13 (72.2)
	S2	–	–	8 (44.4)	10 (55.6)
Felt you had a lot of energy	S1	2 (11.1)	11 (61.1)	5 (27.8)	–
	S2	5 (27.8)	11 (61.1)	1 (5.6)	–
Felt anxious	S1	1 (5.6)	1 (5.6)	6 (33.3)	10 (55.6)
	S2	–	1 (5.6)	9 (50)	7 (38.9)
Felt calm and peaceful	S1	5 (27.8)	10 (55.6)	3 (16.7)	–
	S2	5 (27.8)	10 (55.6)	2 (11.1)	–
Felt enthusiastic about what I was doing	S1	7 (38.9)	11 (61.1)	–	–
	S2	6 (33.3)	10 (55.6)	2 (11.1)	–
Felt guilty	S1	1 (5.6)	1 (5.6)	2 (11.1)	14 (77.8)
	S2	–	–	5 (27.8)	12 (66.7)

S1, Survey 1; S2, Survey 2; *n*, frequency of respondents who gave this answer; %, proportion of respondents who gave this answer; M, mean; SD, standard deviation.

### Competition-related emotions

Competition-related emotions stayed largely the same among the 11 athletes competing in the Warrior Games (Table 6). A greater number of respondents improved than worsened for 6 of the 12 items: “Feel confident,” “Looking forward to competing,” “Concerned may not do as well as could,” “Concerned about performing poorly,” “Concerned others will be disappointed,” “Concerned I won’t be able to concentrate” (Figure 1). However, a greater number of respondents felt more nervous and tense, and less excited at S2 than the number that reported improvements on these items.

**TABLE 6:** Competition-related emotions in Survey 1 and Survey 2 in the 11 athletes competing in the Warrior games.

		Not at all	Somewhat	Moderately so	Very much so
		***n*** **(%)**	***n*** **(%)**	***n*** **(%)**	***n*** **(%)**
Feel nervous	S1^1^	4 (36.4)	3 (27.3)	4 (36.4)	–
	S2^1^	4 (36.4)	3 (27.3)	4 (36.4)	–
Have self-doubts	S1^1^	6 (54.5)	4 (36.4)	1 (9.1)	–
	S2^1^	7 (63.3)	3 (27.3)	–	1 (9.1)
Feel confident	S1^1^	1 (9.1)	1 (9.1)	5 (45.5)	4 (36.4)
	S2^1^	1 (9.1)	1 (9.1)	3 (27.3)	6 (54.5)
Feel tense	S1^1^	5 (45.5)	3 (27.3)	2 (18.2)	–
	S2^1^	2 (18.2)	5 (45.5)	3 (27.3)	–
Concerned I may not do as well in competition as I could	S1^1^	2 (18.2)	4 (36.4)	2 (18.2)	2 (18.2)
	S2^1^	4 (36.4)	3 (27.3)	2 (18.2)	1 (9.1)
Concerned about performing poorly	S1^1^	3 (27.3)	4 (36.4)	3 (27.3)	–
	S2^1^	6 (54.5)	2 (18.2)	1 (9.1)	1 (9.1)
Feel excited	S1^1^	2 (18.2)	2 (18.2)	5 (45.5)	2 (18.2)
	S2^1^	2 (18.2)	7 (63.6)	1 (9.1)	1 (9.1)
Worried about reaching my goal	S1^1^	–	4 (36.4)	4 (36.4)	3 (27.3)
	S2^1^	2 (18.2)	2 (18.2)	3 (27.3)	4 (36.4)
Concerned others will be disappointed in my performance	S1^1^	5 (45.5)	5 (45.5)	1 (9.1)	–
	S2^1^	8 (72.7)	1 (9.1)	1 (9.1)	1 (9.1)
Looking forward to competing	S1^1^	1 (9.1)	2 (18.2)	1 (9.1)	6 (54.5)
	S2^1^	1 (9.1)	1 (9.1)	4 (36.4)	5 (45.5)
Concerned I won’t be able to concentrate	S1^1^	5 (45.5)	3 (27.3)	2 (18.2)	–
	S2^1^	7 (63.6)	2 (18.2)	1 (9.1)	1 (9.1)
Feel supported	S1^1^	–	–	2 (18.2)	8 (72.7)
	S2^1^	–	1 (9.1)	1 (9.1)	9 (81.8)

S1, Survey 1; S2, Survey 2; *n*, frequency of respondents who gave this answer; %, proportion of respondents who gave this answer; M, mean; SD, standard deviation.

^1^Responses are shown for athletes only (*n* = 11).

### Motivational factors

A majority considered the opportunity to raise awareness of the current situation in their country (*n* = 13), to represent their country (*n* = 11), and spend time with other veterans with disabilities (*n* = 8) to be “extremely important” motivational factors (Table 7). In contrast, the opportunity to demonstrate their abilities to others and make friends and family proud appeared less important, with 3 participants (16.7%), respectively, rating these items as “not at all important.”

**TABLE 7:** Motivation for participating in the Warrior games (Survey 1 only).

	Extremely important	Quite important	Not very important	Not important at all
	***n*** **(%)**	***n*** **(%)**	***n*** **(%)**	***n*** **(%)**
Raise awareness of current situation in my country	13 (72.2)	5 (27.8)	–	–
Represent my country	11 (61.1)	5 (27.8)	–	–
Spend time with other veterans with disabilities	8 (44.4)	5 (27.8)	2 (11.1)	2 (11.1)
Be challenged	7 (38.9)	8 (44.4)	1 (5.6)	1 (5.6)
Work toward a goal	7 (38.9)	9 (50.0)	–	1 (5.6)
Make my friends and family proud	5 (27.8)	5 (27.8)	3 (16.7)	3 (16.7)
Make my country proud	4 (22.2)	8 (44.4)	2 (11.1)	1 (5.6)
Improve my physical health	4 (22.2)	13 (72.2)	1 (5.6)	1 (5.6)
Improve my sporting abilities	4 (22.2)	12 (66.7)	2 (11.1)	–
Experience something outside usual routine	4 (22.2)	6 (33.3)	8 (44.4)	–
Demonstrate my abilities to others	3 (16.7)	6 (16.7)	5 (27.8)	3 (16.7)
Travel	3 (16.7)	8 (44.4)	7 (38.9)	–
Take my mind off things	3 (16.7)	7 (38.9)	6 (33.3)	2 (11.1)
Take part in competitive sport	2 (11.1)	4 (22.2)	8 (44.4)	1 (5.6)

*n*, frequency of respondents who gave this answer; %, proportion of respondents who gave this answer; M, mean; SD, standard deviation.

### Perceived sources of support

Support from family (*M* = 5.35, *SD* = 1.54), sports coaches and trainers (*M* = 5.25, *SD* = 1.24), and friends (*M* = 4.88, *SD* = 1.54) were considered most important during preparations for the Games (Table 3). Family elicited the greatest number of “completely important” responses (*n* = 14, 77.8%). Religion or spirituality (*M* = 3.63, *SD* = 2.22), and the sports psychologist (*M* = 3.67, *SD* = 2.09), were considered least important as sources of support, although the same number of respondents (*n* = 5, 27.8%) considered religion and spirituality to be “not at all important” as “completely important.”

## Discussion

Sleep quality and duration were maintained at S2 for the majority of the sample (Table 2). However, mean sleep duration at both timepoints was lower than the current recommendations of 7–9 h for adults (National Center for Chronic Disease Prevention [CPD], 2017). Literature documents the negative impact of active military duty on sleep and circadian patterns (Shattuck et al., 2018), and of sports training camps on both sleep efficiency and sleep duration (Pitchford et al., 2017; Thornton et al., 2017). One study found that, whilst time in bed increased during a training camp for Australian football players, hours of actual sleep did not (Pitchford et al., 2017). It is possible that respondents’ prior participation in active combat and a full week of training had already restricted sleep duration by the time of S1. It should also be noted that several respondents were living with disability; over a third reported a disability that affected mobility, and just under a third reported a VI. Disability in general, and VI specifically (Peltzer and Phaswana-Mafuya, 2017), have often been associated with poorer sleep, including too much or too little sleep and lower subjective sleep quality. This may be of particular significance for the current sample, for whom exposure to a high intensity training programme and different time zones for both training and the upcoming event may have increased the importance of optimal recovery and their overall sleep requirement (Esteves et al., 2019). Indeed, whilst sleep duration did not decrease between the two surveys, some indicated poorer daytime functioning at S2. Whilst physical activity is generally found to enhance sleep (Driver and Taylor, 2000), increased levels of physical activity without a simultaneous increase in sleep duration may have meant that for some, their sleep requirements were not met during this time. The potential negative physical and psychological outcomes of poor sleep have been evidenced elsewhere in athletes, including altered perceptions of exertion (Fullagar et al., 2015), negative impacts on mood and sporting performance (Fullagar et al., 2015), and increased injury risk due to greater daytime dysfunction (Mah et al., 2018).

As with sleep, well-being and mood remained largely the same across the two surveys. There is evidence that personnel involved in military combat often experience guilt, associated with enjoying one’s life whilst comrades might not be so fortunate (Castro et al., 2015). It is easy to envision how such feelings might be fostered within the environment of a UK-based training camp when one’s unit remained engaged in active combat. However, most participants did not feel guilty, or only some of the time at both timepoints. A full and demanding training programme, and participants’ view of the Games as a means of assisting with Ukraine’s war efforts, may have mediated feelings of guilt. In contrast, there was an increase in the number of participants who felt anxious at least “some of the time,” and a decrease in those who felt anxious “none or almost none of the time.” It is perhaps surprising that anxiety did not decrease. Research has evidenced the deteriorations in psycho-emotional status associated with the onset of the ongoing conflict in Ukraine (Kurapov et al., 2022), and the link between combat duties and increased anxiety in military personnel (Pietrzak et al., 2012). Whilst the training camp offered time away from the conflict and any military duties, sports camps are associated with high levels of both physical and mental fatigue (Buchheit et al., 2013). By S2, respondents had spent several weeks away from home, family and comrades. The negative impacts of itinerant work and separation from support networks has been documented in both military and sporting literature (Wiens and Boss, 2006; Dehghansai et al., 2021). It is possible that competition-related emotions may have also influenced anxiety. The majority of respondents indicated that they felt more tense by S2, levels of excitement did not improve for any respondents, and nervousness increased for a greater number than it improved. These emotions may have been heightened due to the immanency of the Games at S2. Whilst participation in veteran sporting activities has been associated with psychological benefits (Laferrier et al., 2017; Walter et al., 2021), such events have also been found to increase stress and negative emotions such as anger, and to decrease positive emotions such as excitement (Roberts et al., 2019). It is notable that more respondents improved on performance-related items (e.g., concerns about doing well and disappointing others). This suggests that, whilst feelings of nervousness and tension were present, overall, the team felt prepared for the Games. Feeling nervous is common in athletes prior to competitions and may, in fact, be useful if managed effectively, offering individuals increased perceptions of control and a focus for positive mental imagery (Wadey and Hanton, 2008).

Despite the physical separation, family was considered the most important source of support during respondents’ preparations. Yet, the expectations of family members in relation to competition outcomes were of little importance. This reflects existing evidence of the grounding role played by family and significant others during periods of training and competition (Kristiansen and Roberts, 2010; Özdemir, 2019) providing a source of love and support that is not contingent on success or performance (Hellstedt, 2005).

Respondents acknowledged the importance of several factors in their decision to participate in the Games, many of which reflected those identified in existing research on veteran sporting events: to improve health and well-being, achieve a goal, and to reconnect with military life (Sporner et al., 2009; Roberts et al., 2021). The opportunity to spend time with other veterans with disability was considered an extremely important factor in several participants’ decisions to attend the Games, further evidencing the value of being able to bond with individuals with similar experiences for attendees at veteran sporting events (Sporner et al., 2009; Roberts et al., 2021). Team Ukraine’s participation in the Warrior Games comes at a unique time in their history, and results suggest that respondents also sought the opportunity to represent their country and raise awareness of the ongoing conflict on an international stage. This highlights the continued role of sporting events, and sports more generally, as socio-political platforms of protest and demonstration (Kaufman and Wolff, 2010).

## Limitations and future research

This paper offers insight into the experiences of a small but unique cohort of Warrior Games attendees. This means that findings may not be generalisable to a broader veteran sample, and the small sample size meant that comparative analysis was not possible. However, it does provide novel insight into the experiences associated with sports participation during times of conflict for veterans and military personnel, and the unique role it might fulfil during this time. Given the importance of participating as a means of raising awareness and representing one’s country in the current study, consideration of the perceptions of other competing teams would be useful in establishing if, and how, Team Ukraine achieved these goals through their participation. Such research would contribute not only to a better understanding of the impact of veterans’ sports participation on the individual, but also the wider political implications of national representation at such events. Exploration of other nations’ experiences of preparing for veteran sporting events may also be valuable in identifying cultural differences in motivational factors, training approach, attitudes, and impacts of participation.

Results indicated increased daytime dysfunction and changes to some aspects of mood and competition-related emotions. However, data pertaining to the period prior to respondents’ arrival in the UK, or during the Games, was not available, and the time between surveys was small. Furthermore, it was not possible to deliver S1 immediately on participants’ arrival in the UK, due to the context of their arrival and the prioritisation of the mental and physical well-being of participants and the start of their training schedule. The current project needed to be designed and delivered within a small timeframe to ensure access to this unique sample population, with continued uncertainty regarding if, and when, team Ukraine would arrive in the UK. Whilst future research which seeks to gather data on health and well-being prior to training camps and beyond participation in sporting events would be beneficial, the realities of doing so with the current sample, many of whom were engaged in active armed conflict prior to the training camp and would return to these duties following the Games, meant that this was not possible in the current study. Future research which considers sleep, and other health and well-being markers, during disabled veterans’ participation in sports and exercise activities, training camps, and competitions, could be used to inform the development of effective schedules of training and rest, ensuring enjoyment, positive health and well-being outcomes, and optimum recovery and performance.

## Conclusion

This study offers insight into the health and well-being of the Ukrainian 2022 Warrior Games team during a UK-based training camp. Results showed that sleep, mood, and competition-related emotions remained largely similar over the course of the camp, although, sleep duration was low at both time points and daytime dysfunction increased, along with feelings of nervousness and tension. This was despite participants indicating that overall, they felt prepared for the Games. Despite the physical separation from loved ones, family was considered an important source of support during the training camp. Respondents perceived the Games as a platform on which to raise awareness of the ongoing war. This demonstrates the role played by sporting events in addressing not only personal goals, but also a shared political agenda. Future research should seek to establish the incidence of, and mechanisms underlying, positive and negative psychological experiences and associated changes in markers of health and well-being prior to, during, and following participation in veteran sporting events. This would help to inform the design of training and support programmes for veteran-athletes, with implications for other amateur and disabled athlete groups.

## Data availability statement

The raw data supporting the conclusions of this article will be made available by the authors, without undue reservation.

## Ethics statement

Ethical review and approval was not required for the study on human participants in accordance with the local legislation and institutional requirements. The patients/participants provided their written informed consent to participate in this study.

## Author contributions

CC and RG conceptualised the study. CC, RG, and NH contributed to study design, methodology, reviewed, and edited the manuscript. CC and NH designed the survey, collected and analysed data, and managed the project. CC wrote the first draft of the manuscript. All authors contributed to the article and approved the submitted version.
